# Enhanced Arboviral Surveillance to Increase Detection of Jamestown Canyon Virus Infections, Wisconsin, 2011–2016

**DOI:** 10.4269/ajtmh.18-0575

**Published:** 2018-12-10

**Authors:** Eduard Matkovic, Diep K. Hoang Johnson, J. Erin Staples, Maria C. Mora-Pinzon, Lina I. Elbadawi, Rebecca A. Osborn, David M. Warshauer, Mark V. Wegner, Jeffrey P. Davis

**Affiliations:** 1 Division of Public Health, Wisconsin Department of Health Services, Madison, Wisconsin;; 2 Department of Pathology and Laboratory Medicine, University of Wisconsin School of Medicine and Public Health, Madison, Wisconsin;; 3 Arboviral Diseases Branch, Division of Vector-Borne Diseases, Centers for Disease Control and Prevention, Fort Collins, Colorado;; 4 Department of Medicine, University of Wisconsin School of Medicine and Public Health, Madison, Wisconsin;; 5 Career Epidemiology Field Officer, Office of Public Health Preparedness and Response, Centers for Disease Control and Prevention, Atlanta, Georgia;; 6 Wisconsin State Laboratory of Hygiene, Madison, Wisconsin

## Abstract

Jamestown Canyon virus (JCV), a mosquito-borne *Orthobunyavirus* (within the California serogroup), can cause severe neuroinvasive disease. According to national data during 2000–2013, 42% of the 31 documented JCV disease cases in the United States were detected in residents from Wisconsin. The Wisconsin Division of Public Health enhanced JCV surveillance by implementing routine use of JCV-specific immunoglobulin M (IgM) antibody testing followed by confirmatory JCV-specific plaque reduction neutralization testing on all patients with suspected cases of arboviral infection who had tests positive for arboviral immunoglobin at commercial laboratories. During 2011–2016, of the 287 Wisconsin specimens tested on the Arbovirus IgM Antibody Panel, 30 JCV cases were identified (26 confirmed and four probable). Twenty-seven (90%) JCV cases were detected after 2013. Among all cases, 17 (56%) were male and the median age was 54 years (range: 10–84 years). Fifteen patients had neuroinvasive disease, including meningitis (*n* = 9) and meningoencephalitis (*n* = 6). Although historically considered rare, the relatively high rate (0.12 cases/100,000 population) of diagnosis of JCV infections among Wisconsin residents during 2013–2016 compared with that in previous years suggests occurrence is widespread throughout Wisconsin and historically may have been under-recognized. This study aims to raise awareness of JCV infection for differential diagnosis among the arboviral diseases. Improved and timely diagnosis of arboviral disease is important in that it will provide more information regarding emerging infections and promote preventive measures to avoid mosquito-borne exposure and infection among residents of and visitors to affected areas.

## Introduction

Jamestown Canyon virus (JCV) is a mosquito-borne *Orthobunyavirus* within the California serogroup initially isolated during 1961 from a pool of *Culiseta inornata* mosquitoes from Jamestown Canyon, Colorado.^1^ Jamestown Canyon virus is widely distributed throughout temperate North America and has been isolated in at least 26 species of mosquitos; *Aedes* and *Ochlerotatus* species are the primary vectors in the Midwestern United States.^2–8^ Viral transmission occurs through the bite of an infected mosquito.

Human JCV infection was originally described during the 1960s as a cause of minor illness among forest workers in Wisconsin.^9^ This perception remained until 1982, when JCV was identified as the causal agent of moderate to severe central nervous system disease, most common among adults.^10^ Although human JCV cases are rare, neuroinvasive disease, such as meningitis or meningoencephalitis, is detected among 54–79% of reported cases, and often requires hospitalization.^11,12^ Apart from supportive and symptomatic management, there are no specific treatments for or vaccines available to prevent JCV infection.

During 2011, the Wisconsin Division of Public Health (WDPH) initiated testing for JCV in its surveillance activities as part of the enhanced arboviral disease surveillance. In 2013, arboviral surveillance activities were further enhanced with more routine and comprehensive testing for evidence of JCV infection because of an increase in available funding and resources. We describe the implementation of Wisconsin’s enhanced arboviral surveillance program as focused on JCV infections and the clinical and epidemiologic features of JCV disease reported among Wisconsin residents.

## Methods

### Enhanced surveillance activities.

During 2011, a passive surveillance system was used at WDPH to detect JCV among the California serogroup viruses. The surveillance consisted of some commercial laboratories sending specimens with positive tests for immunoglobulin (Ig) M antibody to La Crosse virus (LACV) or other California serogroup viruses to Wisconsin State Laboratory of Hygiene (WSLH) for repeat serology testing. As arboviral tests may produce false-positive results between unrelated viruses or cross-reactive results between viruses in the same family, genus, or serogroup, all IgM-positive or equivocal WSLH test results were forwarded to the Arboviral Diagnostic Laboratory. Specimens forwarded to the Centers for Disease Control and Prevention (CDC) underwent diagnostic algorithms using IgM antigen capture enzyme-linked immunosorbent assay (MAC-ELISA), immunoglobulin G (IgG) ELISA, IgM microsphere immunoassay, and plaque reduction neutralization test (PRNT). Confirmed laboratory results included a 4-fold or greater change in JCV antibody titer in paired sera and identification of JCV IgM antibody in cerebrospinal fluid (CSF) or serum (acute or convalescent) with appropriate confirmatory testing (positive PRNT). The PRNT was based on the methods described by Lindsey et al.^13^ that measured virus-specific neutralizing antibody titers.^14,15^ Positive JCV IgM ELISA with PRNT titer ≥ 10 and other arboviral PRNT titers were < 10 was considered to be evidence of recent JCV infection. For samples with a positive JCV IgM but with PRNT titers ≥ 10 for JCV and another arbovirus not in the California serogroup, the results were interpreted as a coinfection as long as IgM was positive for the second virus for which PRNT titers were ≥ 10. If a sample was JCV IgM positive by ELISA and had PRNT titers ≥ 10 for both JCV and LACV, the JCV titer had to be more than 4-fold higher than the LACV titer to be considered to be evidence of recent JCV infection. Differential neutralization testing for California serogroup viruses did not include snowshoe hare virus (SSHV) as LACV is more closely related to SSHV than JCV and is more likely to cause cross-reactive titers, as was previously noted.^16,17^ Probable laboratory results included positive JCV IgM antibody in an acute or convalescent serum specimen without performing PRNT for confirmation. No evidence of JCV infection (negative) was reported if JCV PRNT titers were < 10 in samples collected ≥ 7 days after illness onset. Furthermore, serum samples positive for only IgG were suggestive of previous exposure to a mosquito-borne virus.

To increase JCV case detection, the WDPH transitioned into an active surveillance system during 2013 by initiating routine requests—for confirmatory testing at CDC—of specimens positive for LACV IgM and other California serogroup viruses from commercial laboratories. Concurrently, WDPH further enhanced its surveillance by requesting that results of all commercially tested sera positive for arboviral IgG or IgM antibody be reported to WDPH. Testing included a virus-specific MAC-ELISA^18^ and PRNT on all IgM-positive serum or CSF specimens.

In 2016, WSLH validated a JCV-specific IgM MAC-ELISA to help improve detection and expedite results. All specimens directly submitted for arbovirus testing and all specimens with positive arboviral tests from commercial laboratories underwent screening at the WSLH for Eastern equine encephalitis virus, Powassan virus (POWV), LACV, and JCV using a virus-specific IgM MAC-ELISA, and for West Nile virus (WNV) and St. Louis encephalitis virus using a microsphere immunoassay; all results were confirmed at CDC.^19^

### Case detection.

During 2011–2016, follow-up investigations were conducted for positive commercial-test results to determine whether the patient had a clinically compatible arboviral illness (e.g., fever, myalgia, meningitis, and encephalitis). The investigation of each suspected case was conducted using a standardized arbovirus case report form. Laboratory-confirmed cases were cross-checked with clinical information reported in medical records. Cases of JCV disease were defined using the Council of State and Territorial Epidemiologists (CSTE) case definitions for arboviral diseases, neuroinvasive and non-neuroinvasive, which defines a case of arboviral disease as meeting clinical criteria and one or more of the laboratory criteria (Table 1).^20^ Confirmed cases were reported to electronic surveillance systems including the Wisconsin Electronic Disease Surveillance System and CDC ArboNET.

**Table 1:** Case definitions of Jamestown Canyon virus disease using an adaptation of the CSTE case definitions of arboviral disease

Clinical criteria
* Neuroinvasive disease*: requires the presence of the following:
Fever (≥ 100.4°F or 38°C) as reported by the patient or a health-care provider
* *Meningitis, encephalitis, acute flaccid paralysis, or other acute signs of central or peripheral neurologic dysfunction, as documented by a physician
* *Absence of a more likely clinical explanation
* Non-neuroinvasive disease* requires the presence of the following:
* *Fever (≥ 100.4°F or 38°C) as reported by the patient or a health-care provider
* *Absence of neuroinvasive disease
* *Absence of a more likely clinical explanation
Laboratory criteria for confirmed case included either of the following:
* *Isolation of virus from, or demonstration of specific viral antigen or nucleic acid in, tissue, blood, CSF, or other body fluid
* *Four-fold or greater change in virus-specific quantitative antibody titers in paired sera
* *Virus-specific IgM antibody in serum with confirmatory virus-specific neutralizing antibody titers in the same or a later specimen
* *Virus-specific IgM antibody titers in CSF and a negative result for the other IgM antibody in CSF for arboviruses endemic to the region where exposure occurred
Laboratory criteria for probable case
* *Virus-specific IgM antibody in CSF or serum.
* *Probable
* Neuroinvasive disease:* A case that meets the above clinical criteria for neuroinvasive disease and the following laboratory criteria: Virus-specific IgM antibody in CSF or serum but with no other testing.
* Non-neuroinvasive disease:* A case that meets the above clinical criteria for non-neuroinvasive disease and the laboratory criteria for a probable case: Virus-specific IgM antibody titers in CSF or serum but with no other testing.
* *Confirmed
* Neuroinvasive disease:* A case that meets the above clinical criteria for neuroinvasive disease and one or more of the following laboratory criteria for a confirmed case listed above.
* Non-neuroinvasive disease:* A case that meets the above clinical criteria for non-neuroinvasive disease and one or more of the following laboratory criteria for a confirmed case listed above.

CSF = cerebrospinal fluid; CSTE = Council of State and Territorial Epidemiologists; IgM = immunoglobulin M.

### Data collection and analysis.

For all probable and confirmed JCV disease cases, data were collected regarding residence, gender, age, onset of exposure, mosquito bite and outdoor exposure, travel history, past medical history (e.g., immunodeficiency), signs and symptoms, laboratory data, history of blood transfusion and organ transplantation, hospitalization, length of hospital stay, discharge status, and mortality. Information was gathered on clinical signs and symptoms related to neurological manifestations, in addition to clinical syndromes associated with meningoencephalitis, meningitis, encephalitis, and acute febrile illness.

Study variables were analyzed using Excel spreadsheet (Microsoft version 15.33; Microsoft Corp., Redmond, WA) and Data Desk version 6.3 (Data Description, Inc., Ithaca, NY). Continuous variables were reported as a median with an associated range, and categorical variables were presented as counts and frequencies. All cases identified were included in the analysis. Comparative data analysis evaluated intervals leading up to and following 2013 when enhanced JCV surveillance was implemented. Chi-square and F-tests were used to examine differences in case characteristics and clinical features between patients with neuroinvasive and non-neuroinvasive disease. Data from cases with a coinfection were compared with those cases with evidence of only JCV infection. Using 2011–2015 census data from Wisconsin Interactive Statistics on Health and Wisconsin Department of Administration to measure urbanicity, counties were placed into one of three population density groups: high (> 100 persons per square mile), medium (40–99 persons per square mile), and low (< 40 persons per square mile).^21,22^ Mean annual incidence rates per 100,000 persons were calculated over the study period using the cumulative average of the total population of Wisconsin and of each Wisconsin county over the study period (Table 2).

**Table 2:** Mean annual reported incidence of Jamestown Canyon virus disease incidence by population density (persons per square mile) among the 20 counties with one or more reported cases, Wisconsin, 2011–2016

Population density: persons/mile^2^	No. of cases	No. of counties	Total population	Annual incidence*
Low: < 40	12	32	695,852	1.72
Medium: 40–99	6	19	990,301	0.60
High: > 100	12	21	4,080,421	0.30

* Annual incidence per 100,000 population.

## Results

**Figure 1.**
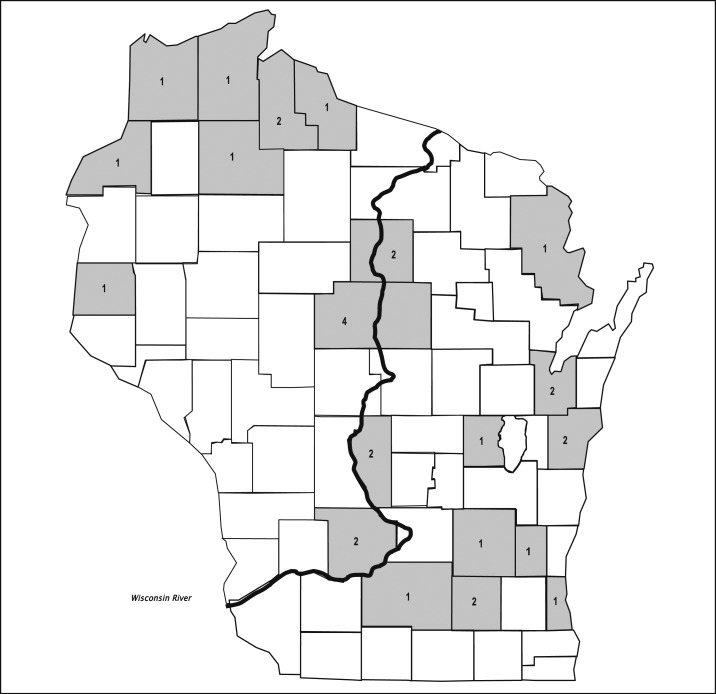
Number of confirmed and probable cases of Jamestown Canyon virus disease (*n* = 30) by county of residence, Wisconsin, 2011–2016.

During 2011–2016, 30 cases of JCV disease (26 confirmed and four probable) were identified in Wisconsin residents (Figure 1). Before implementation of enhanced surveillance efforts during 2013, only three cases of JCV disease had ever been detected by the WDPH. During 2013–2016, 27 cases were detected, including 12 during 2013, five during 2014, four during 2015, and six during 2016. The mean annual reported incidence of JCV disease in Wisconsin increased about 4.5-fold from 0.03 cases/100,000 population during 2011–2012 to 0.12 cases/100,000 population during 2013–2016.

Of 30 case patients, six (20%) case patients had evidence of another acute arboviral infection (four had evidence of confirmed or probable WNV and two with POWV) and 20 (67%) had test results that showed cross-reactivity, false-positive IgM results that did not confirm with PRNT, or a previous infection (i.e., IgG antibodies) with another arbovirus (LACV or WNV). Coinfection was confirmed in four patients with neuroinvasive disease (two WNV and two POWV coinfections) and two with a non-neuroinvasive disease (two WNV coinfections).

### Geographical distribution.

The 30 patients with JCV disease reported during 2011–2016 were residents of 20 Wisconsin counties (Figure 1). These included 22 (73%) residents of eastern and central counties of Wisconsin, among whom nine resided in counties that border the Wisconsin River. Eight (27%) patients resided in northwestern Wisconsin counties and no cases occurred among residents of southwestern Wisconsin counties. Among the 20 counties with at least one reported case during 2011–2016, the nine with low population densities had a greater aggregated mean annual reported incidence (1.7 cases per 100,000 population) than the six counties with medium population densities (0.6/100,000 population) and five counties with high population densities (0.3/100,000 population).

### Epidemiologic features.

**Figure 2.**
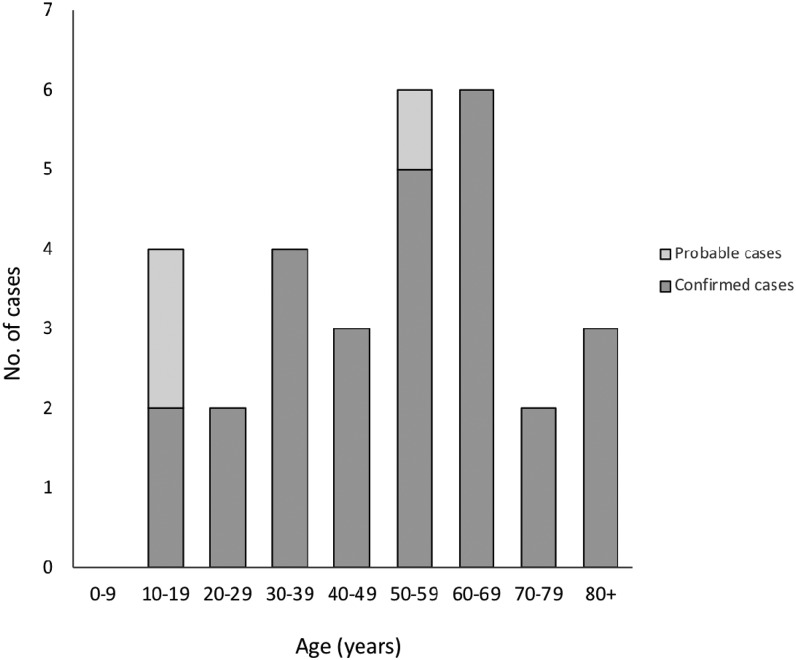
Cases (confirmed and probable) of Jamestown Canyon virus disease by age, Wisconsin, 2011–2016 (*n* = 30).

**Figure 3.**
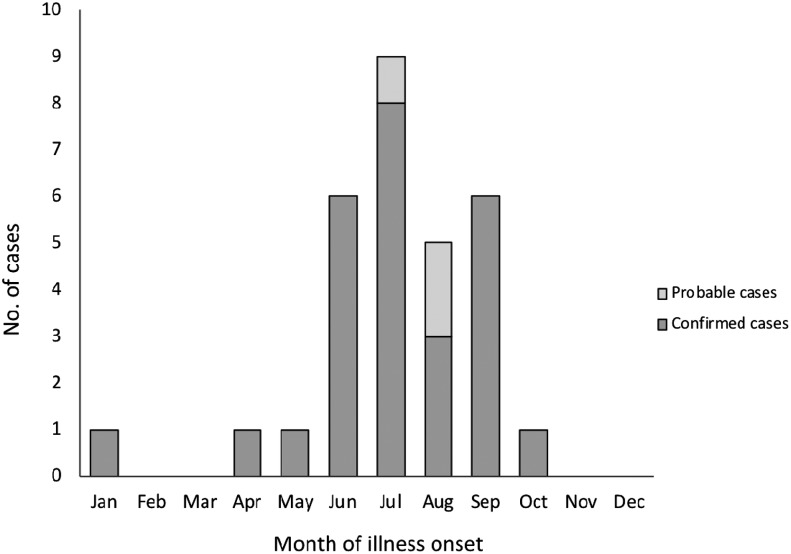
Cases (confirmed and probable) of Jamestown Canyon virus disease by month of illness onset, Wisconsin, 2011–2016 (*n* = 30).

Among the 30 patients with JCV disease, 17 (56%) were male, the median age was 54 years (range: 10–84 years), and only four (13%) patients were aged < 20 years (Figure 2). Outdoor exposure was reported by 22 (73%) patients, and 17 (56%) patients reported mosquito bites. Twenty-four (80%) patients had no travel history outside of Wisconsin during the 14 days before illness onset, indicating that exposure to infected mosquitoes most likely occurred within Wisconsin counties (Table 3). Fifteen (50%) patients had neuroinvasive disease and 15 (50%) patients had non-neuroinvasive disease. Among the case characteristics examined, patients with neuroinvasive disease differed significantly from patients with non-neuroinvasive disease only in percentage hospitalized (Table 3). Patients with neuroinvasive disease did not differ in age or gender when compared with patients with non-neuroinvasive disease. Illness onset occurred more frequently during the summer months; 87% of patients had onsets during June through September and cases peaked during July (Figure 3). The demographics, location, and clinical syndrome for the six patients with coinfections did not differ significantly from the 24 with evidence of only JCV infection.

**Table 3:** Characteristics of patients with reported neuroinvasive and non-neuroinvasive JCV disease, Wisconsin, 2011–2016 (*n* = 30)

Case characteristics*	Total cases (*n* = 30)	Neuroinvasive cases (*n* = 15)	Non-neuroinvasive cases (*n* = 15)
Median age (years)	54	51	56
Meningitis cases	–	45	–
Encephalitis cases	–	60	–
Age range (years)	10–84	10–80	10–84
Males, no. (%)	17 (56)	9 (60)	8 (53)
Outdoor exposure, no. (%)	22 (73)	12 (80)	10 (66)
History of mosquito bite, no. (%)	17 (56)	9 (60)	8 (53)
No travel outside state, no. (%)	24 (80)	14 (93)	10 (66)
Hospitalized, no. (%)†	14 (47)	11 (73)	3 (20)
Mechanical ventilation, no. (%)	3 (10)	3 (20)	0 (0)
Discharge to LTAC/SNF/NH, no. (%)	5 (17)	4 (26)	1 (6)
Median duration of hospital stay (days)	6	7	3
Range of hospital course (days)	2–38	2–38	2–7
Died, no. (%)‡	1 (3)	1 (6)	0 (0)

JCV = Jamestown Canyon virus; LTAC = long-term acute care; NH = nursing home; SNF = skilled nursing facility.

* For six patients, data collection was incomplete for hospitalization, ventilation, length of hospital stay, discharge to LTAC/SNF/NH, travel history, mosquito bite, or outdoor exposure.

† *P* < 0.05 (comparison between neuroinvasive and non-neuroinvasive cases).

‡ Coinfection case with West Nile virus and JCV.

### Clinical and laboratory features.

Fifteen (50%) patients had neuroinvasive disease including meningitis (*n* = 9) and meningoencephalitis (*n* = 6). Meningitis was detected more frequently among a younger age group than meningoencephalitis (Table 3). Among all signs and symptoms examined, only the occurrence of balance disturbances was found to be significantly more frequent in neuroinvasive disease than in non-neuroinvasive (Table 4). Patients with neuroinvasive disease were hospitalized at a higher rate than those with non-neuroinvasive disease, although median duration of hospital stay was similar (6–7 days). Patients with neuroinvasive disease were discharged to long-term care at a higher rate than those with non-neuroinvasive disease. Patients with neuroinvasive disease experienced central neurological manifestations including headache, neck rigidity, altered mental status, gait/balance disturbances, dizziness, seizures, or tremors. No focal neurological deficits were recorded (Table 4). Three patients with neuroinvasive disease required mechanical ventilation (Table 3). A separate comparison of the six cases with coinfection between the remaining JCV cases revealed no significant differences in clinical findings when factoring for neuroinvasive and non-neuroinvasive disease (*P* = 0.36). There was one death in a patient with neuroinvasive disease; however, we cannot attribute this finding to JCV as the patient was coinfected with WNV. The patient was an 80-year-old female with a clinical diagnosis of meningoencephalitis, exhibiting signs and symptoms of altered mental status, gait disturbance, and slurred speech with elevated CSF counts and hypernatremia. Initial arboviral tests were presumptively IgM positive for LACV, JCV, and WNV; PRNT confirmed recent infection with JCV and WNV.

**Table 4:** Clinical manifestations of neuroinvasive and non-neuroinvasive Jamestown Canyon virus disease, Wisconsin, 2011–2016 (*n* = 30)

Signs and symptoms*	Total cases number (%) (*N* = 30)	Neuroinvasive disease number (%) (*N* = 15)	Non-neuroinvasive disease number (%) (*N* = 15)
Fever			
Documented	25 (83)	12 (80)	13 (86)
Unknown	5 (17)	3 (20)	2 (14)
Generalized weakness	21 (70)	12 (80)	9 (60)
Headache	20 (66)	12 (80)	8 (53)
Myalgia	18 (60)	9 (60)	9 (60)
Nausea	11 (37)	6 (40)	5 (33)
Neck rigidity	9 (30)	7 (46)	2 (14)
Altered mental status	8 (23)	7 (46)	1 (6)
Balance disturbances†	6 (20)	6 (38)	0 (0)
Dizziness	5 (16)	4 (26)	1 (6)
Photophobia	4 (13)	3 (20)	1 (6)
Tremors	4 (13)	3 (20)	1 (6)
Arthralgia	4 (13)	3 (20)	1 (6)
Dysarthria	3 (10)	3 (20)	0 (0)
Memory deficit	2 (6)	2 (13)	0 (0)
Seizures	2 (6)	2 (13)	0 (0)

* Body temperature was unknown for five patients whose data collection was incomplete (two non-neuroinvasive and three neuroinvasive cases). Although chills were reported in three of these patients, these cases are classified as *unknown* for fever. In nine patients, data collection was incomplete for laboratory values and physical examination with signs and symptoms.

† *P* < 0.05 (comparison between neuroinvasive and non-neuroinvasive cases).

The following two inflammatory markers were elevated in 15 patients; the median erythrocyte sedimentation rate was 64 mm/hour (range: 12–88 mm/hour) and the median C-reactive protein (CRP) level was 26 mg/L (range: 2.2–101 mg/L). Although one patient presented with hypernatremia (148 mmol/L), no other significant abnormalities in serum electrolytes were detected. Among nine patients with available imaging data, four patients with neuroinvasive disease had magnetic resonance neuroimaging suggestive of viral infection. Results demonstrated signal intensity in gray matter of bilateral hemispheres, basal ganglia, and dural thickening. Results of electroencephalography studies were either unremarkable or nonspecific. Among nine patients with neuroinvasive disease who had available CSF results, six cases had lymphocytic pleocytosis (median: 52 cells/mm3; range: 39–6,050 cells/mm^3^) with protein elevation (median: 76 mg/dL; range: 56–362 mg/dL). Cerebrospinal fluid white cell counts were greater among patients with meningitis than in patients with encephalitis and elevated neutrophils (range: 32–1,808 cells/mm^3^) in CSF were observed in three patients. Patients with evidence of coinfections were more likely to have elevated CSF counts (lymphocytic pleocytosis: median, 101 cells/mm^3^ and protein level: median, 117 mg/dL) and CRP levels (median, 61 mg/L), and imaging studies were abnormal in two cases with neuroinvasive disease.

## Discussion

Enhanced surveillance efforts, including testing for JCV infections among persons with evidence of arboviral IgG or IgM antibody and compatible clinical signs and symptoms to an arboviral infection, has substantially increased the number of JCV cases detected in Wisconsin. During 2011–2016, the 30 cases of JCV disease reported among Wisconsin residents represent more than half of all cases of JCV disease reported in the United States during that interval.^2,23^ Although it is unclear whether the small number of cases reported during 2011–2012, the initial 2 years of Wisconsin’s JCV surveillance, was related to limited JCV transmission, results of the current enhanced surveillance suggest that human JCV infection occurs more frequently than previously thought.

Neuroinvasive disease was identified among 50% of the patients, a lower percentage than previously reported.^2^ This could have resulted from increased testing and identification of JCV disease among individuals with mild illness or the potential detection of antibodies formed following a recent asymptomatic infection. Wisconsin residents infected with JCV were less likely to have encephalitis than meningitis, a finding which is supported by previous studies.^2^ Substantial morbidity was associated with neuroinvasive disease, reflected by the need to hospitalize 73% of such patients. However, because four patients with neuroinvasive disease had a coinfection with another arbovirus, some clinical signs and symptoms and the overall morbidity might have been attributable to or worsened by the other infection. Symptoms of JCV infection are difficult to differentiate from those caused by LACV, which is also endemic in Wisconsin. In comparison to LACV, however, our JCV cases demonstrated that children were less frequently affected, with no cases in children less than 10 years of age and 63% of cases aged > 40 years.^24–27^

The geographic range of JCV is one of the widest among California serogroup viruses,^28^ with a slight predilection for more northern states.^2^ Enhanced JCV surveillance methods provided a more refined understanding of the geographic distribution. It remains unclear whether the discrepancy in reporting is a result of underreporting or an established geographic niche in Wisconsin. Among Wisconsin counties with reported cases, higher incidence rates were associated with less densely populated counties. Fewer cases were detected within counties with population densities greater than 150 persons per square mile. Rural areas and suburbs, with their typically wooded properties and increasing human populations, are likely important habitats for interaction between people, environment, and arthropod vectors.^29^

The wide distribution might be additionally attributed to JCV remaining endemic among white-tailed deer and other free-ranging ungulates living in larger areas with flat topography. Jamestown Canyon virus was the most seroprevalent of the three California serogroup viruses (LACV, JCV, and Trivittatus) examined among Wisconsin’s white-tailed deer; 77% of the deer sampled had neutralizing antibodies to the virus.^30^ In addition, JCV has been identified in 26 species of mosquitoes with different ecological breeding patterns. Although not all species might be efficient vectors of JCV transmission, the fact that several of these species are commonly found to be infected in nature, including *Aedes*, *Ochlerotatus*, *Culex*, *Anopheles*, and *Culiseta* species,^31,32^ suggests JCV might have a diverse and potentially broad transmission pattern. Certain mosquitoes (e.g., *Aedes stimulans*) may be responsible for transmitting JCV earlier during the year, whereas other species may transmit JCV later, creating a bimodal peak in the seasonal distribution of JCV disease.^28^ Transovarian transmission may also be a contributing factor in early seasonal occurrence. The range of vectors potentially involved in JCV transmission makes environmental control strategies difficult. In addition, not all the mosquitoes are of woodland type; therefore, avoiding infested areas (wooded area, wetlands, and river banks) alone might not be an ideal preventive measure. In addition to mosquito diversity, most cases of JCV disease in Wisconsin occurred in the summer months (June to September) during periods of active mosquito transmission, and one case was detected in January, suggesting that a life cycle independent of mosquitoes can lead to year-round detection.^30^

Our study has several limitations. Medical records review was restricted to inpatient and discharge records; therefore, no information regarding long-term outcome postinfection was obtained. This is particularly relevant for those who had neuroinvasive disease. Moreover, not all hospital charts were complete. Fever or vital signs were not documented in five patients, possibly resulting in misclassification of patient diagnoses. Ultimately, the small number of cases limits generalization of our conclusions. Six of the patients, including four with neuroinvasive disease, tested positive for more than one arbovirus. The contribution of JCV to clinical presentation among these patients is unclear. We also did not perform differential neutralization testing for SSHV relying on LACV PRNTs to note potential cross-reactivity. This could have led to cases being incorrectly classified as JCV versus SSHV. Finally, it is possible that the IgM and neutralizing antibodies detected in some individual’s samples might represent previous asymptomatic infection. This might explain a confirmed case reported in January when there is little to no mosquito activity in Wisconsin. Furthermore, this could lead to misclassification and potentially misrepresentation of the signs and symptoms being attributable to JCV infections.

Although there is no specific treatment for JCV disease, obtaining a timely diagnosis can help with determining clinical management or avoiding unnecessary procedures. Improved and timely arboviral disease diagnosis is important to provide more information regarding emerging infections and promoting preventive measures to avoid exposure and infection among residents and visitors of the affected areas.

## Conclusion

Although historically considered rare, the relatively high rate of diagnosis of JCV disease among Wisconsin residents compared with rates noted elsewhere in the United States suggests the JCV disease occurrence is widespread throughout Wisconsin and affects a wide range of age groups. The increase in detection of JCV infection and disease in Wisconsin was more likely the result of enhanced surveillance that incorporated sensitive diagnostic methods and complete reporting, rather than the emergence of JCV. Jamestown Canyon virus infections have been likely underreported, which might result from lack of commercially available JCV-related testing, misdiagnosis resulting from cross-reactivity with other arboviruses, and lack of awareness among medical providers and other health professionals. Our enhanced surveillance efforts likely improved diagnosis of JCV disease, increased JCV disease recognition, and decreased rates of false-positive IgM results for related viruses such as LACV in Wisconsin. Through enhanced efforts, surveillance in Wisconsin was better able to quantify the burden and public health impact of JCV disease.

## References

[1] Calisher CH, 1983 Taxonomy, classification, and geographic distribution of California serogroup bunyaviruses. Prog Clin Biol Res 123: 1–16. 6346334

[2] Pastula DM Hoang Johnson DK White JL Dupuis AP Fischer M Staples JE, 2015 Jamestown Canyon virus disease in the United States-2000–2013. Am J Trop Med Hyg 93: 384–389. 26033022 10.4269/ajtmh.15-0196 PMC4530766

[3] Clark GG Crabbs CL Watts DM Bailey CL, 1986 An ecological study of Jamestown Canyon virus on the Delmarva Peninsula, with emphasis on its possible vector. J Med Entomol 23: 588–599. 2879043 10.1093/jmedent/23.6.588

[4] Hardy JL Eldridge BF Reeves WC Schutz SJ Presser SB, 1993 Isolations of Jamestown Canyon virus (Bunyaviridae: California serogroup) from mosquitoes (Diptera: Culicidae) in the western United States, 1990–1992. J Med Entomol 30: 1053–1059. 7903698 10.1093/jmedent/30.6.1053

[5] Smith GC Moore CG Davis T Savage HM Thapa AB Shrestha SL Karabatsos N, 1993 Arbovirus surveillance in northern Colorado, 1987 and 1991. J Med Entomol 30: 257–261. 8433334 10.1093/jmedent/30.1.257

[6] Walker ED Grayson MA Edman JD, 1993 Isolation of Jamestown canyon and snowshoe hare viruses (California serogroup) from *Aedes* mosquitoes in western Massachusetts. J Am Mosq Control Assoc 9: 131–134. 8350066

[7] Centers for Disease Control and Prevention CDC Arboviral Catalog, 2016 *Jamestown Canyon Virus*. Available at: https://wwwn.cdc.gov/arbocat/VirusDetails.aspx?ID=206&SID=2. Accessed December 6, 2016.

[8] DeFoliart GR Anslow RO Hanson RP Morris CD Papadopoulos O Sather GE, 1969 Isolation of Jamestown canyon serotype of California encephalitis virus from naturally infected *Aedes* mosquitoes and tabanids. Am J Trop Med Hyg 18: 440–447. 5768778 10.4269/ajtmh.1969.18.440

[9] Thompson WH, Evans AS, 1965 California encephalitis virus studies in Wisconsin. Am J Epidemiol 81: 230–244. 14261029 10.1093/oxfordjournals.aje.a120511

[10] Grimstad PR Shabino CL Calisher CH Waldman RJ, 1982 A case of encephalitis in a human associated with a serologic rise to Jamestown Canyon virus. Am J Trop Med Hyg 31: 1238–1244. 7149109 10.4269/ajtmh.1982.31.1238

[11] Srihongse S Grayson MA Deibel R, 1984 Claifornia serogroup viruses in New York state: the role of subtypes in human infections. Am J Trop Med Hyg 33: 1218–1227. 6507732 10.4269/ajtmh.1984.33.1218

[12] Grimstad PR, 2001 Jamestown Canyon virus. Service MD, ed. Encyclopedia of Arthropod- Transmitted Infections of Man and Domestic Animals. New York, NY: CABI Publishing, 235–239.

[13] Lindsey HS Calisher CH Mathews JH 1976 Serum dilution neutralization test for California group virus identification and serology. J Clin Microbiol 4: 503–510. 1002829 10.1128/jcm.4.6.503-510.1976 PMC274511

[14] Russell PK Nisalak A, 1967 Dengue virus identification by the plaque reduction neutralization test. J immunol 99: 291–296. 4961907

[15] Russell PK 1967 A plaque reduction test for dengue virus neutralizing antibodies. J immunol 99: 285–290. 6031202

[16] Kosoy O 2016 Serological survey for antibodies to mosquito-borne bunyaviruses among US National Park service and US forest service employees. Vector Borne Zoonotic Dis 16: 191–198. 26855300 10.1089/vbz.2015.1865

[17] Hughes HR Lanciotti RS Blair CD Lambert AJ, 2017 Full genomic characterization of California serogroup viruses, genus orthobunyavirus, family peribunyaviridae including phylogenetic relationships. Virology 512: 201–210. 28985574 10.1016/j.virol.2017.09.022

[18] Martin DA Muth DA Brown T Johnson AJ Karabatsos N Roehrig JT, 2000 Standardization of immunoglobulin M capture enzyme-linked immunosorbent assay for routine diagnosis of arboviral infections. J Clin Microbiol 38: 1823–1826. 10790107 10.1128/jcm.38.5.1823-1826.2000 PMC86599

[19] Wisconsin Department of Health Services, 2016 *Arboviral Disease-Laboratory Guidance*. Available at: https://www.dhs.wisconsin.gov/arboviral/labguide.htm. Accessed December 9, 2016.

[20] Centers for Disease Control and Prevention, 2011 *Arboviral Dis Neuroinvasive Non-Neuroinvasive, 2014 Case Definition*. Available at: http://wwwn.cdc.gov/nnds/conditions/arboviral-diseases-neuroinvasive-and-non-neuroinvasive/case-definitions/2011/. Accessed January 6, 2017.

[21] Wisconsin Interactive Statistics on Health, 2016 *Population Estimates*. Available at: https://www.dhs.wisconsin.gov/population/index.htm. Accessed January 5, 2017.

[22] Sotir MJ Glaser LC Fox PE Doering M Geske DA Warshauer DM Davis JP, 2007 Endemic human mosquito-borne disease in Wisconsin residents, 2002–2006. WMJ 106: 185–190. 17844707

[23] United States Geological Survey, 2017 *Vector-Borne Diseases and Zoonotic Diseases Archive*. Available at: https://archive.usgs.gov/archive/sites/health.usgs.gov/vector_zoonotic/. Accessed August 15, 2017.

[24] Mayo D Karabatsos N Scarano FJ Brennan T Buck D Fiorentino T Tran S, 2001 Jamestown Canyon virus: seroprevalence in Connecticut. Emerg Infect Dis 7: 911–912. 11747714 10.3201/eid0705.017529 PMC2631874

[25] Gaensbauer JT Lindsey NP Messacar K Staples JE Fischer M, 2014 Neuroinvasive arboviral disease in the United States: 2003 to 2012. Pediatrics 134: 642–650. 25113294 10.1542/peds.2014-0498 PMC5662468

[26] Hunt WG McJunkin JE, 2014 La Crosse encephalitis and other California serogroup viruses. Cherry J Demmler-Harrison GJ Kaplan SL Steinbach WJ Hotez P, eds. Feigin and Cherry’s Textbook of Pediatric Infectious Diseases, 7th edition Philadelphia, PA: Elsevier, 2513–2537.

[27] Lindsey NP Lehman JA Staples JE Fischer M; Division of Vector-Borne Diseases, National Center for Emerging and Zoonotic Infectious Diseases, CDC, 2014 West Nile virus and other arboviral diseases–United States, 2013. MMWR Morb Mortal Wkly Rep 63: 521–526. 24941331 PMC5779373

[28] Grimstad PR, 1988 California group virus disease. Monath TP, ed. The Arboviruses: Epidemiology and Ecology. Boca Raton, FL: CRC Press, 99–136.

[29] Falco RC Wormser GP Dainels TJ, 2008 Suburbanization in developed nations. *The Social Ecology of Infectious Diseases*. 1st edition. Mayer KH, Pizer HF, eds. Amsterdam, The Netherlands: Elsevier Inc. 5:138–170.

[30] Issel CJ Trainer DO Thompson WH, 1972 Serologic evidence of infections of white-tailed deer in Wisconsin with three California group arboviruses (La Crosse, Trivittatus, and Jamestown Canyon). Am J Trop Med Hyg 21: 985–988. 4635779 10.4269/ajtmh.1972.21.985

[31] Andreadis TG Anderson JF Armstrong PM Main AJ, 2008 Isolations of Jamestown Canyon virus (Bunyaviridae: *Orthobunyavirus*) from field-collected mosquitoes (Diptera: Culicidae) in Connecticut, USA: a ten-Year analysis, 1997–2007. Vector Borne Zoonotic Dis 8: 75–88. 10.1089/vbz.2007.0169 18386967

[32] Armstrong PM Andreadis TG, 2007 Genetic relationships of Jamestown Canyon virus strains infecting mosquitoes collected in Connecticut. Am J Trop Med Hyg 77: 1157–1162. 18165540

